# Non-monotonic dynamics and crosstalk in signaling pathways and their implications for pharmacology

**DOI:** 10.1038/srep11376

**Published:** 2015-06-18

**Authors:** Roeland van Wijk, Sander J. Tans, Pieter Rein ten Wolde, Alireza Mashaghi

**Affiliations:** 1 FOM Institute AMOLF, Science Park 104, 1098 XG Amsterdam, The Netherlands; 2 Department of Bionanoscience, Kavli Institute of Nanoscience, Delft University of Technology, Lorentzweg 1, 2628 CJ Delft, the Netherlands; 3 Harvard Medical School, 25 Shattuck St, Boston, MA 02115, USA

## Abstract

Currently, drug discovery approaches commonly assume a monotonic dose-response relationship. However, the assumption of monotonicity is increasingly being challenged. Here we show that for two simple interacting linear signaling pathways that carry two different signals with different physiological responses, a non-monotonic input-output relation can arise with simple network topologies including coherent and incoherent feed-forward loops. We show that non-monotonicity of the response functions has severe implications for pharmacological treatment. Fundamental constraints are imposed on the effectiveness and toxicity of any drug independent of its chemical nature and selectivity due to the specific network structure.

In pharmacology and toxicology the effect of a drug is typically described by a monotonic (linear or non-linear) dose-response curve^1^. For monotonic dose-response relations, the effectiveness reaches its maximum at a certain dose, and increasing the dose beyond this point does not (significantly) increase the effectiveness further. At this dose-saturation point, the maximum effectiveness depends on the chemical nature of the drug with respect to the target choice. Assuming the most appropriate target for drug treatment is chosen, improvements of the effectiveness of the treatment can be explored by focusing on the selectivity of the drug for the target. Indeed, a long-standing dogma in pharmacology is that selectivity implies effectiveness and safety. Therefore, in order to maximize the effectiveness (while minimizing side effects), rational drug design traditionally has been focused on the discovery of maximally selective and potent drugs^2^. Significant investments are made by pharmaceutical industry in the synthesis and screening of a large number of potential drugs, aiming for higher effectiveness and lower toxicity.

Structural analysis of biochemical networks helps in target selection thereby improving effectiveness and toxicity. The choice of the target is an important determinant of the resulting effectiveness and toxicity. A significant step forward in rationalizing the choice of drug targets was realized following advances in molecular biology and network theory^3^,^4^,^5^,^6^. In recent years, many components of signaling pathways and their interactions are discovered and represented using detailed knowledge of the molecular connections within the signaling network^7^,^8^. These developments allow for selecting targets on a stronger rational basis. For example, bridging nodes, that connect modular subregions of the signaling network, are promising drug targets from the standpoints of effectiveness and side effects, since their disruption would specifically prevent information flow between network modules of interest (high effectiveness) while it does not lead to any global change in the network (low toxicity)^9^. In the case of antibiotics and anti-cancer drugs, (network) hub nodes are considered interesting targets, and indeed for commercialized antibiotics and anti cancer drugs, the average number of interaction partners for protein targets are 4 and 8 respectively^10^.

A key question is whether network dynamics and topology reveal constraints on the form of the pharmacological response and provide insights into toxicity and effectiveness^11^,^12^,^13^,^14^. If the network analysis could provide additional insight into the network dynamics, on the form of the dose-response relation, on fundamental limits of achievable effectiveness and possible toxicity, and on how to administer the designed drugs, it would significantly reduce the cost of the drug development. How the form of the dose-response relation depends on the network topology and dynamics has remained to be systematically explained. One fundamental characteristics of the dose-response relation is its (non)monotonicity. While a monotonic form has been typically assumed in pharmacology, there is growing evidence that many bio-molecular pathways have non-monotonic dose-response relationships^15^,^16^,^17^,^18^,^19^ with potential functional significance. An example of its use is transmitting different biochemical signals through one and the same pathway, and yet respond to them specifically—a phenomenon that is called multiplexing in telecommunication and computer networks^20^.

To address this fundamental question, it is instructive to consider small signaling networks with a specific topology, investigate the pharmacological implications of such a network topology, and evaluate the advantages and limitations of the selective (and non-selective) pharmacology approaches. A simple, yet general and dynamically rich example would entail two linear signaling pathways with inter-pathway interactions. Canonical signaling pathways in cells are known to interact with each other via direct regulation or via their shared components (cross-talk)^21^. Large-scale surveys of inter-pathway interactions, revealed extensive crosstalk between pathways^22^,^23^. Cross-talk between signaling pathways results in non-linearity and dynamical monotonicity in response to stimuli due to mechanisms that are not well understood^23^.

These, and other topological interactions, though important^24^, are not the only crucial ingredient in the functioning of signaling networks. Indeed, the topological structure by itself not necessarily dictates the dynamics of the network^25^; the dynamical behavior is determined by the kinetic parameters of the network as well. Moreover, cells are able to multiplex signals along pathways. For example, the highly conserved MAPK (Mitogen Activated Protein Kinase) pathway is predicted to be one such pathway with multiplexing capabilities^26^. The pathway includes an incoherent feed-forward loop involving Ras-Raf-Akt. The design of drugs that target components of a MAPK pathway is an area of intense research^20^,^27^,^28^,^29^.

In this article we take two linear signaling pathways, allow for inter-pathway interactions (both shared components and biochemical interactions) and determine the input-output relations for many different topologies. Then, for each topology we look for interactions and dynamic parameters that generate a non-monotonic input-output relation, providing mechanistic insight into the origin of the non-monotonicity. Next, to study the effect of non-monotonicity on effectiveness and toxicity of pharmacological approaches we study two simple examples of two linear pathways, with either direct interaction and/or a shared component. We study a network with two inputs and two outputs where one pathway directly regulate the other pathway and study the occurrence of non-monotonicity and its effect on drug treatment in detail. In the remainder we describe the fundamental constraints, due to the non-monotonicity of the dose-response relationship, on the effectiveness and toxicity depending on the target choice of each network. We find that therapeutic windows for certain therapies are narrow and the preferred concentration range might occur after a broad toxic regime.

## Non-monotonicity in simple networks

**Figure 1:**
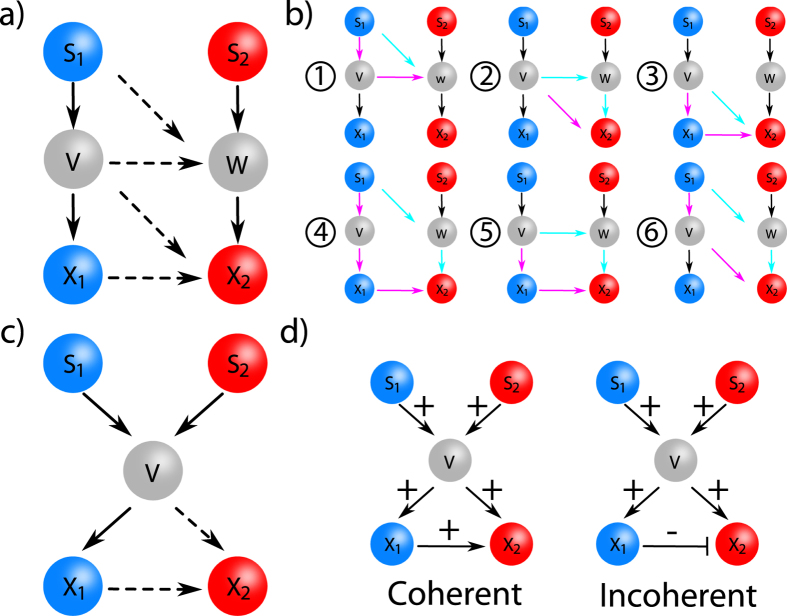
Overview of networks. Each network consists of two pathways S1 → X1 (Left) and S2 → X2 (Right). The pathways may interact via regulatory interactions and a shared component. Solid arrows are either activating or repressive, while dashed arrows can be activating, repressive or absent. **a**) 6 node topology. **b**) 6 different possible incoherent feed-forward loops marked by colored arrows (magenta activating, light blue repressing) **c**) 5 node topology. **d**) example of an incoherent and coherent feed-forward loop for a 5 node topology (+ activating, − repressing).

We start by studying two simple networks to show the theoretical abundance of non-monotonic behavior in networks consisting of two signaling pathways which share components and have biochemical interactions. For this we take two 3-node signaling pathways. Both signaling pathways have one input signal (S_1_,S_2_, respectively) and one response (X_1_,X_2_, respectively) each. Here the simplest interaction is a direct interaction between a node from one path and a node from another path. This is represented in the first network consisting of 6 nodes (Fig. 1a). In this network S_1_ affects both X_1_ and X_2_, but S_2_ only affects X_2_. A less trivial interaction arises when both signaling pathways share the same node. This is investigated in the other network, consisting of 5 nodes (Fig. 1c), where both signals influence both responses. In both networks we exclude direct regulation of any signal to either response. This corresponds to common processes, e.g. extracellular signals are transmitted via a receptor to the intracellular domain, or intracellular signals are transmitted to the DNA in the nucleus via messenger proteins.

Each response is either in an active form 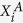 or inactive form *X*_*i*_. We model the interactions between the different components using the well-known Monod-Wyman-Changeux (MWC) model (see Methods), which has shown to agree with experiments on proteins^30^,^31^,^32^. The MWC model is a statistical-physical model for the probability of a protein to be in its active state, which depends both on the intrinsic energies and on the binding of other molecules. Before we proceed, we formally have to define a non-monotonic response. We imagine each response to be in one of three states, ON, OFF, NONE, with the following definition


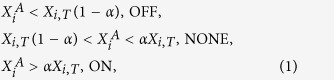


When the level of the active form of components, 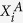, is higher than a fraction *α* > 0.5 of the total concentration of a specific component *i*, *X*_*i*,*T*_, i.e. if 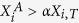, then the response is considered ON. Similarly if 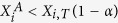 it is considered OFF. In the intermediate ramp, 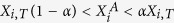, the response is neither OFF nor ON; this is the NONE state. This state is included to take into account that in the presence of noise, an intermediate response may not lead to a significant phenotypic response at the cell population level. We note however, that for *α* = 0.5, there is no NONE state and that none of the conclusions presented below depend on the precise choice of alpha.

As an example we describe a non-monotonic response of X_1_ as function of S_2_. This is defined as a double change in the state of X_1_, ON-OFF-ON or OFF-ON-OFF due to an increase or decrease in S_2_, while S_1_ is kept at a constant level.

It is often recognized that non-monotonicity is the result of incoherent feed-forward loops; and this can be explained qualitatively, both for dynamical and steady-state responses^33^,^34^. For this reason in the 5 and 6 node networks we focus on the influence of (in)coherent feed-forward loops on possible steady-state non-monotonic behavior. A feed-forward topology consists of three components (A,B,C, where A regulates B directly and C via B. In a coherent feed-forward loop the regulation of B and C by A are equal (e.g. both activating), while in an incoherent loop the regulations are opposite (activating and repressing).

### 6-node topology

The 6-node network (Fig. 1a) consists of two linear pathways S_1_ → X_1_ (Left) and S_2_ → X_2_ (Right). Further, from the left cascade (S_1_ → X_1_) at various positions regulatory interactions to the right (S_2_ → X_2_) cascade can be present. We exclude interactions from the right to the left pathway to prevent bistability in the results. We sample parameters over all possible topologies (*N* = 2^4^ × 3^4^ = 1296) (see Supporting Material). For this 6-node network, 6 different incoherent feed-forward loops could be present, depending on the sign of each interaction arrow (Fig. 1b). In total, 800 out of 1296 topologies have at least one incoherent feed-forward loop, while each incoherent feed-forward loop is present in 288 topologies.

**Figure 2:**
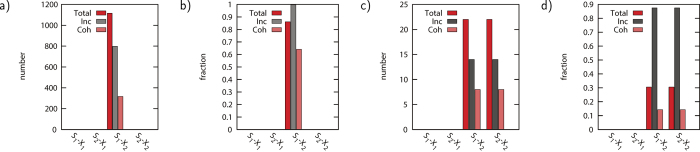
Histogram with the appearance of non-monotonicity for the 5 and 6 node network specified for each combination S_*i*_ − X_*j*_. **a**,**b**) 6 node network, *α* = 0.55, *n* = 2, minimum number of samples >25000. **c**,**d**) 5 node network, *α* = 0.55, *n* = 2, minimum number of samples 47430. **a**,**c**) Number of topologies that show non-monotonicity. **b**,**d**) Fraction of topologies that show non-monotonicity with respect to total number of all/incoherent/coherent topologies (see Fig. 1).

In Fig. 2a,b the number of topologies that have a non-monotonic response are shown, and we split the results into topologies that have an incoherent feed-forward loop and topologies that do not have this. The first observation is that non-monotonic interactions only occur for changes in S_1_ that lead to changes in X_2_. The absence of the other three possible non-monotonic responses can be easily explained. First, S_2_ has no regulatory effect on X_1_ and therefore a non-monotonic S_2_-X_1_ response is not possible. Second, S_1_ to X_1_ is a linear cascade with only one species acting onto another, excluding non-monotonicity between S_1_ and X_1_. An equivalent reasoning holds for the cascade S_2_-X_2_. Although there are more difficult interactions present, for any given value of the components S_1_, V and X_1_, a linear cascade between S_2_ and X_2_ remains. Almost all of the 800 (798) topologies with at least one incoherent feed-forward loop have at least one parameter set that leads to non-monotonic behavior (see SI).

Unexpectedly, we see that not only topologies with incoherent feed-forward loops show non-monotonicity, but also topologies that have only coherent interactions. This effect depends on the specific parameters within the topology, and requires an in-depth explanation. As an example we take a coherent feed-forward loop with a specific set of parameters that lead to non-monotonic behavior. X down-regulates Z, but up-regulates Y, while Y down-regulates Z. Further Z is active, in the absence of both X and Y. Importantly, the binding and activation strength of X and Y for Z are not equal; Y has both a much smaller binding affinity to Z and a much larger down-regulating affect on Z compared to X. Let us discuss the dose-response of Z following an increase in X. Without X present (and thus no presence of Y) Z is active. An increase in X leads to an increase in Y and a decrease in Z. The upregulation of Y by X saturates at a given concentration of X. If Y saturates while X still increases, then X becomes the dominant negative regulator of Z. Since the negative regulation of X is much smaller than that of Y, Z increases again, hence giving rise to a non-monotonic response (see SI for an illustration).

Although coherent feed-forward loops can lead to non-monotonic behavior, non-monotonic behavior is more likely to occur for incoherent feed-forward loops. This can be observed from the fraction of different topologies that show non-monotonic behavior, as well as from the number of parameter sets for each topology that show non-monotonic behavior (see SI).

One can analytically prove that for non-monotonicity it is required that a component is simultaneously regulated by two other components, of which the steady-state value of one of these components depends non-linearly on the other component (see SI).

### 5-node topology

In the 5 node network (Fig. 1c) the component V is shared between the pathways S_1_ → X_1_ and S_2_ → X_2_. In total 72 topologies can be constructed. X_1_ is only regulated by V, and non-monotonic behavior for X_1_ can therefore be excluded. There exists only a single possibility for an incoherent feed-forward loop, which is present if the regulatory effect of the pathway V → X_1_ → X_2_ is opposite to that of the pathway V → X_2_, leading to 16 topologies that have an incoherent feed-forward loop.

In Fig. 2c,d we show histograms of the number (and fraction) of topologies that leads to non-monotonic behavior. We first observe that, as expected, non-monotonic behavior is only observed for X_2_. Out of the possible 72 topologies, 22 show non-monotonicity in the response X_2_ as function of S_1_, and 22 are non-monotonic as function of S_2_.

Not every topology with an incoherent feed-forward loop shows non-monotonicity. This does not imply that non-monotonic behavior for these topologies is impossible. It does show, however, that for these topologies the fraction of parameters within the total sampling space that allow for non-monotonic behavior is so small that brute force sampling did not reveal these (see SI). We again note that there are coherent topologies that show non-monotonicity.

The networks studied here can be seen as networks of interactions between species, but can also be viewed as coarse-grained representations of more complex networks. Specifically, a link between two nodes can be interpreted as an interaction between two species, but it may also represent a linear pathway. The input-output relation of a linear pathway—in the absence of interactions with other pathways—is monotonic and can thus be coarse-grained. Indeed, this coarse-graining does not affect the (non)-monotonicity of a multi-pathway network. Based upon our simple networks, with coherent feed-forward loops, incoherent feed-forward loops and/or shared nodes, we show that these networks can show non-monotonic dose-response relations. We next study the implications of non-monotonicity for pharmacodynamics of two example topologies.

## Pharmacological implications of non-monotonicity

Before proceeding, we first introduce two important quantities: effectiveness and toxicity. *Effectiveness* is quantified as the ability to change the state of a selected response by applying a drug, while *toxicity* is quantified as unwanted change in the state of any other non-selected response. Throughout the paper we assume that the responses X_1_ and X_2_ are non-targettable and therefore a network can only be targeted at the signal level (S_1_, S_2_) or the intermediate components (V,W). Then, by definition, an effective agonist (antagonist) for S_1_ or V increases (decreases) the concentration of S_1_ or V to such a level, that a desired change of the response X_1_ is observed.

We are interested in the effect of drugs that specifically change the state of one and only one signal output (effectiveness) without affecting the state of the other output (toxicity).

### 6-node network

We first select one 6 node network that has a single incoherent feed-forward loop (Fig. 1b. 3); the activating connections S_1_ − V, S_2_ − W, V − X_1_ and X_1_ − X_2_, while V and W both repress X_2_.

**Figure 3:**
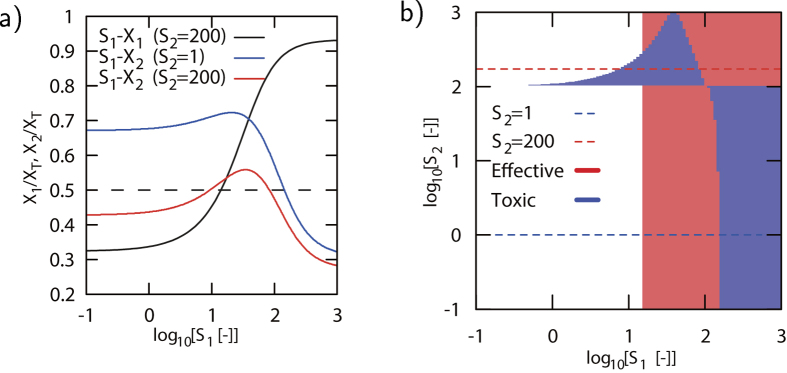
Analysis of non-monotonicity in the 6 node network from Fig. 1b. 3 and its pharmacological implications. *α* = 0.5, *n* = 2.**a**) Dose-response curve for X_1_ as function of S_1_ for S_2_ = 200 (black solid), and X_2_ as function of S_1_ for S_2_ = 200 (red solid) and of S_1_ for S_2_ = 1 (blue solid), showing the non-monotonic response for X_2_ as function of S_1_. **b**) Contour plot showing the toxicity and effectivity of the state of X_2_ as function of an agonist for S_1_ for various concentrations of S_2_. The two horizontal dashed lines at S_2_ = 200 and S_2_ = 1 compare to the curves in **a**).

This network, for specific parameters, displays a non-monotonic relation between S_1_ and X_2_. To illustrate this behavior, Fig. 3a shows the dose-response curves of S_1_ − X_1_ for S_2_ = 200 [a.u.] (black solid), S_1_ − X_2_ for S_2_ = 200 (red solid) and S_1_ − X_2_ for S_2_ = 1 (blue solid). The non-monotonic behavior of X_2_ as a function of S_1_ is clearly observed if concentration S_2_ = 200, in contrast to the case where S_2_ = 1. The consequences of this non-monotonic behavior for pharmacology are shown in Fig. 3b. In this contour plot the effective concentration regime for an agonist for S_1_ is shown (red shading). As expected, a minimum concentration is required to activate X_1_, but due to the network structure there is no dependence on S_2_. The toxic region (blue shading) has a completely different shading. Since the effect of an agonist for S_1_ is studied, toxicity is defined as a change in the state of X_2_ compared to the state of X_2_ in the absence of S_1_, but for equal concentration of S_2_. For the two cases S_2_ = 1,200 in Fig. 3a this means a toxic response occurs when crossing the black dashed line *α* = 0.5, changing the state of X_2_ with respect to its initial state. This is further illustrated by drawing the corresponding red and blue dashed lines in Fig. 3b for S_2_ = 1,200. Fig. 3b shows that at large concentrations of S_2_ (>100) the non-monotonic response leads to a toxic concentration range at intermediate concentrations. A small window exist for which the drug becomes toxic, before it is effective. Interestingly, due to the non-monotonic response, the drug becomes non-toxic and effective at even larger concentrations. At low concentrations of S_2_ the toxic regime occurs at large agonist concentrations, and the drug is effective before a toxic effect occurs.

### Effect of intrinsic differences between individuals

In the previous paragraph we showed that specific drugs are toxic for this 6-node network. From a pharmaceutical perspective, not only the toxicity as function of total dose, but also as a function of intrinsic fluctuations in network kinetics is relevant. In this section we study this in more detail.

It is well known that inter- and intra-individual variations exist for kinetic parameters due to variations in physicochemical properties of the internal environment, like the temperature, pH and concentration of components^35^. For example, despite the fact that human body temperature is tightly controlled, human body temperature varies between healthy individuals for nearly a degree centigrade^36^. This variation has significant implications for kinetics, as the rate constants depend exponentially dependent on temperature. Indeed, one can estimate based upon temperature changes that differences up to ≈10% are possible in the rate constants. Similarly enzymatic reaction rates depend on pH^37^. In the following, we study the effect of variations in the kinetic parameters in general and investigate their effect on toxicity and effectiveness.

Since our analysis depends on specific dissociation constants (e.g. between S_1_ and V), we here set out to study the influence of these variations on the effectiveness and toxicity of an antagonist. As in the previous paragraph, we study the effect of an agonist for S_1_, assuming (the red dashed line in Fig. 3b). Additionally, we study the effect of an agonist for V, assuming S_1_ = 0.1 and S_2_ = 200. To mimic the intrinsic variations in the dissociation constants, we vary each parameter (*P*) (see SI) in the system following


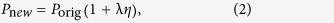


where *η* is a random number sampled from a uniform distribution between [−1:1] and λ is the maximal percentage change in each parameter. As a result of this procedure, each parameter *P*_n*ew*_ is bounded between *P*_n*ew*_[*P*_o*rig*_(1 − λ):*P*_o*rig*_(1 + λ)].

**Figure 4:**
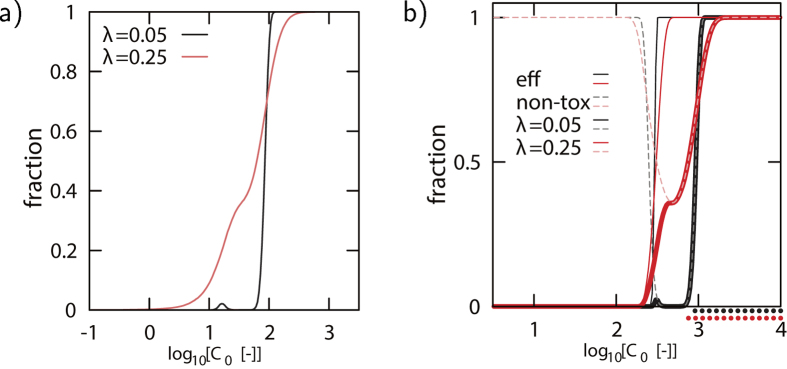
The effect of intrinsic variability in the 6 node network from Fig. 1b. 3. *α* = 0.5, *n* = 2. A relative change λ in all the intrinsic parameters (see Eq. (2)) leads to a reduction of the effectiveness and/or an increase in the toxicity. Shown is the fraction of parameter combinations for which a drug with concentration *C*_0_ is effective and non-toxic. **a**) Lines show for an agonist for S_1_ (with S_2_ = 200) the fraction of individuals for which—at the given dose *C*_0_—an effective and non-toxic treatment is obtained. **b**) Similar results for an agonist for V, however the results for effectiveness (thin lines) and non-toxicity (thick lines) are shown separately. The solid thick lines indicates the dose for which the drug is non-toxic and effective. The dotted thick lines denotes the therapeutic window (S_1_ = 0.1,S_2_ = 200).

The current analysis provides a way to determine the therapeutic window as a function of the variation variable λ. The therapeutic window of a drug is the range of drug concentrations that elicits a therapeutic response without undesired toxic effects. It is quantified as the drug range between the ED_50_ and the TD_50_ point, where TD_50_ denotes the dose at which toxic response is observed in 50% of the population and ED_50_ denotes the dose at which a therapeutic response is observed in 50% of the population. The results are shown in Fig. 4.

An agonist for S_1_ (Fig. 4a) requires for increasing λ (increasing the variability) an increase in minimum drug concentration for a 100% effective and non-toxic dose. In other words, a higher variability leads to a larger concentration that is effective for all individuals. Interestingly, the concentration for which only a small fraction of individuals has an effective and non-toxic treatment increases. This is due to the fact that larger differences between individuals exist, and the probability that a relative low concentration works for a single individual increases. As a result, the therapeutic window is larger for larger variability. In Fig. 4b a similar dependence on the variability for an agonist for V is shown. Here for two values of λ both the effectiveness (solid thin) and non-toxic (dashed thin) fractions as function of the agonist concentration are shown, together with the fraction of both effective and non-toxic simultaneously (solid thick). The non-monotonic response is clearly observed by following the non-toxicity line. The fraction of simultaneous effective and non-toxic (solid thick) for low concentration rises because of the increase in the effectiveness, but is then limited by the increase of toxicity. Only when the toxicity decreases again, can the drug become both effective and non-toxic. The therapeutic window (below the graph,thick dotted)), shows that for larger variability the window is larger. Note that the steepness of the curves means that a small change in the curve can imply a large difference in the fraction of non-toxic and effective cells (see SI).

### A 5-node network: the multiplexing topology

**Figure 5:**
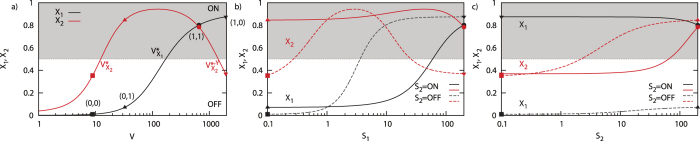
Dose-response curves for X1 (black), X2 (red) as a function of **a**) V, **b**) S1, and **c**) S2 for the incoherent topology in Fig. 1d. Shading indicates the regime for which the response X is ON, assuming α = 0.5. **a**) The dose-response curve for X1 (black) and X2 (red) as function of V. Further the critical concentrations 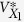, 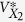, 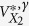 are indicated where the state switches from ON to OFF, or vice versa. The symbols indicate the state of the input signals, ■ = (*S*_1_ = OFF, *S*_2_ = OFF), ▲ = (*S*_1_ = OFF, *S*_2_ = ON) ● = (*S*_1_ = ON, *S*_2_ = OFF) ▼ = (*S*_1_ = ON, *S*_2_ = ON) (see Eq. 3). The non-monotonic relation between X2 and V is clearly observed. **b**) The dose-response curve for for X1 (black) and X2 (red) as function of S_1_. Solid lines indicate S_2_ = ON, dashed lines S_2_ = OFF. The non-monotonic relation between X2 and S1 is clearly observed. **c**) Similar to **b**), but with the roles of S_1_ and S_2_ reversed.

The multiplexing topology is the incoherent 5-node network as shown in Fig. 1d. Downstream of V an incoherent feed-forward loop exist, where V activates X_1_ and X_2_, while X_1_ represses X_2_. This network has a shared node (V) and an incoherent feed-forward loop. A closer look at this topology reveals a non-monotonic relation between X_2_ and S_1_ and between X_2_ and V (see Fig. 5). Here we study the effects of these non-monotonic relations on the effectiveness and toxicity of drugs.

The most important result is that a specific drug could effectively lead to a change in the response of X_*i*_, but as a result also change the state of the other response X_*j*≠*i*_; leading to a drug that is effective but toxic. Importantly, normally drugs are applied in a gradual manner, and, upon the desired change in a specific response the treatment is stopped. At this drug concentration however, the treatment could be toxic. More intriguing, a toxic response could occur before the effective response is observed, or the toxicity could disappear for higher concentrations.

For a specific parameter set, this network is capable of multiplexing signals^20^. This indicates that the response X_1_ reflects the state of S_1_ independent of the state of S_2_, and similarly for X_2_. Since we have two binary input signals, we can define four specific input patterns,


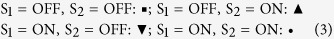


Assume S_1_ and S_2_ are both in the OFF state, and we target S_2_ with an agonist. As a result, X_2_ switches from OFF to ON, and at the moment of switch, X_1_ has not changed (see Fig. 5c, starting from the ■). This agonist therefore is effective and non-toxic. Similarly, an agonist for S_1_, while X_2_ is OFF, switches X_1_ from OFF to ON (Fig. 5b). But now, at the moment of the transition X_2_ has changed state as well. The agonist therefore is effective, but toxic. A further increase of agonist would eventually remove the toxicity, while remaining effective (see Fig. 5b, starting from ■). This is a direct consequence of the non-monotonic relation between S_1_ and X_2_.

Lastly, we discuss drugs for the intermediate component V, showing an interesting discrepancy between an agonist and antagonist. Assume we start in the state where S_1_ and X_1_ are OFF, S_2_ is ON and X_2_ is ON (see Fig. 5a, ▲) and we would like to use a drug that switches X_2_ from ON to OFF. Applying an antagonist for V, decreasing V drives the network to state ■. During this process, X_2_ switched from ON to OFF, while X_1_ has not changed state. The antagonist is thus effective and non-toxic. On the contrary, an agonist for V, driving the network to ▼-state, leads to an effective, but toxic response, since then both X_1_ and X_2_ change state. The direction of driving in this example determines the toxicity of the drug, but does not change the effectiveness. Again, this is an immediate consequence of the non-monotonic relation between V and X_2_, combined with the initial position of the system. We summarize the results in Table 1 (see SI for complete table).

So far we have assumed that we can change a single component (e.g. 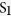) by applying a drug, while the other component(s) remain(s) constant. This assumption of course is not necessarily valid. If the other signal is non-constant, effective drug-doses can become ineffective. Imagine that starting in a state with both 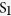 and 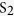 in the ON state (● in Fig. 5a), we add antagonist for 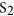 which drives 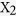 to the OFF-state (▼). If 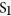 is not constant in the ON-state because, for example, it has a circadian rhythm, then there can be periods during the circadian cycle where the antagonist is non-effective, since during the gradual oscillations of 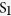 from ON to OFF (state ▼ to ■), the response 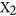 switches from OFF to ON to OFF.

#### Influence of agonist dose and administration interval

In the previous paragraph we showed that specific drugs are toxic for this 5-node network with multiplexing capacity. From a pharmaceutical perspective, not only the toxicity as function of total dose, but also as a function of dose-interval is interesting. In this paragraph we study this in more detail.

Consider the case with initial state ■ and final state ▲, where we provide an agonist for S_2_ (see Fig. 5c). In the previous section we have shown that this treatment is effective and non-toxic, but this observation changes if we consider the dynamics of drug administration. For a typical clinical treatment, we imagine that the average agonist concentration equals the required effective concentration *C*_0_. However, for a non-continuous uptake and some type of degradation/dilution of agonist over time, this implies that the actual agonist concentration varies as a function of time (see Methods).

Importantly, it is not the average dose *C*_0_, but the actual dose *C*(*t*) (see Eq. (13)) that determines the toxicity and effectiveness of the drug. Indeed, for such a delivery process we need to specify toxicity and effectiveness in more detail. A drug is effective, if both for the minimal and maximal dose of the drug (thus for the full dose-range of the protocol) the required effectiveness is observed (e.g. X_2_ from OFF to ON). A drug is considered toxic if within the dose-interval (at least at the minimal or at the maximal drug dose) a toxic effect is observed.

In the remaining part of this section we discuss the dependence of toxicity and effectiveness on the required dose *C*_0_, the time between administrations *τ* and the dilution rate *k*.

**Figure 6:**
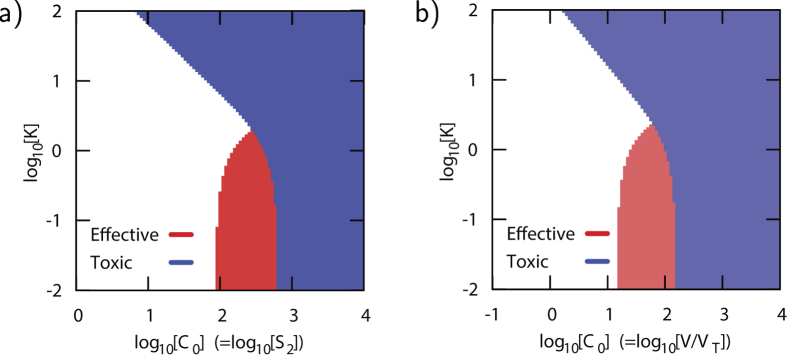
**a,b**) Contour plots for the effectiveness (red shading) an toxicity (blue shading) as function of average dose *C*_0_ and administration parameter *K* = *kτ* (see Fig. 7) for a system that **a**) is initially in state ▼ (see Fig. 5) with an agonist for S_2_, and **b**) is initially in state ■ with an agonist for V. Parameters: *α* = 0.55, *n* = 2.

In Fig. 6a we show the effectiveness and toxicity of an agonist for S_2_ as a function of *C*_0_ and *K* = *kτ* for a network that initially is in state ▼ (S_1_ is ON and S_2_ is OFF). Applying an agonist for S_2_ to the system from state ▼ leads to a decrease in the concentration *V* affecting both X_1_ and X_2_. To switch X_2_ from OFF to ON a minimal drug concentration is required. Indeed, in Fig. 6a, we observe that for too low average concentrations *C*_0_ no change in X_2_ is observed (absence of red shading), leading to a lower bound on the minimum concentration (see Eq. (15)) for an effective treatment. Further, for too large average concentrations *C*_0_ at *K* or relatively small average concentrations at large *K* the drug is toxic (blue shading). The red shaded regime shows the “total dose-interval”-space for which the agonist is always effective (X_2_ OFF to ON), and never toxic (X_1_ remains ON).

Instead of targeting S_2_, one could also directly target V, in order to change X_2_. An agonist for V, trying to bring the system from state ■ to state ▲, has a very narrow window of effectiveness (Fig. 6b). This narrow window originates from the different interaction between V and X_2_, and X_1_ and X_2_. The increase in the concentration *X*_2_ by increasing concentration *V* is suppressed by a an increase in the concentration *X*_1_, which in turn represses X_2_. Since for a large agonist dose *C*_0_, X_1_ is pushed from the OFF into the ON state, this concentration is toxic.

These results show that it is possible that a drug for which the required dose *C*_0_ is non-toxic, if administered at constant levels , can become toxic, depending on the administration procedure.

#### Polypharmacology strategies

In the preceding discussions, the drug was assumed to be selective for a single-target, resulting in the exploration of single-target therapeutic strategies. We discussed effective, non-toxic pharmacological protocols for different scenarios. In some cases, we observed that single-target therapies are inherently toxic (see Table 1). For these scenarios, we study the effect of a multi-target approach, following the concept of polypharmacology. Recently evidence has emerged indicating that the effectiveness of a significant number of approved drugs is rooted in their promiscuity^38^,^39^,^40^,^41^,^42^. These observations led to the development of polypharmacology. In practice however, the paradigm of polypharmacological drug design has intrinsic difficulties: developing a drug that influences several targets in a desired way is a tedious and expensive task 43. Thus any prior knowledge about the achievable effectiveness and toxicity would be highly valuable.

Here we ask if polypharmacological approaches can provide a non toxic therapeutic strategy when selective therapy is toxic. We focus on the case where we would like to use an agonist for S_1_ to drive X_1_ from OFF to ON; we thus would like to bring the system from state ■ to state ▲, a situation we have shown to be toxic if an agonist for S_1_ is administered. From a polypharmacological perspective, besides targeting S_1_ the drug can target both V and S_2_, either in a antagonistic or agonistic fashion. This creates four different scenarios.

Unfortunately, in all scenarios the non-selective therapy remains toxic. The origin of the toxicity lies in the ordering of the so called critical concentrations, e.g. the critical concentration 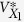 is the concentration *V* for which X_1_ switches state. Since, by construction of the multiplexing motif, 
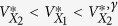
 (see Fig. 5a), at the first transition to the OFF-state for X_1_, X_2_ is in the ON-state, while it initially was in the OFF-state. For a gradual increase in the drug, this toxicity can not be overcome, even if polypharmacologic drugs are considered.

Here we explain the response of the current topology to polypharmacologic drugs. If the agonist for S_1_ would also act as an agonist for V, the result would be that for a smaller concentration of agonist the toxic response is observed. If the drug would act as agonist for S_1_ but an antagonistic for V, either no effective response is observed (if the antagonistic effect is too strong), or an effective response is observed, yet the drug is also toxic. Even worse, this polypharmacologic drug can be toxic, but not effective, if the concentration *V* is decreased below 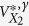, but not below 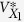. Similar arguments can be formulated for an agonistic or antagonistic effect of the drug with respect to S_2_. In any case, due to the ordering of the critical values V^*^, the drug is toxic at the crossing of 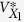. Clearly, for this topology, polypharmacological approaches would not overcome the toxicity.

## Conclusion

In this study we have discussed the occurrence of non-monotonicity in interacting signaling pathways in the context of drug targeting. We have shown that non-monotonic input-output relations can arise not only in incoherent feed-forward loops, but also in coherent feed-forward loops. We note that network topologies that contain coherent and incoherent feed-forward loops, do not necessarily show non-monotonicity. Binding and activation of molecular components are found to critically influence the form of the response function as well.

The described non-monotonicity is often left unnoticed experimentally. Our study considered a network topology, which incorporates features (incoherent feed-forward, multiple ligand activation) that are commonly observed in many cellular processes, which has a non-monotonic dose-response relation. The state of the art high-throughput methods for generating dose-response curves, namely robotic microplate-based and microfluidics-based approaches, construct dose-response relation based on small datasets^44^,^45^,^46^. In these analyses therefore, delicate non-monotonic features in dose-response curves are probably left undetected. With the emergence of high-resolution methods we expect to increasingly discover complex response functions^47^,^48^.

Non-monotonicity imposes fundamental constraints on the pharmacodynamics of a drug as well as the choice of administration routes and protocols, irrespective of the chemical nature of the designed drug. The models discussed in this article are very simple in nature, and by construction we know all its parameters. Even though our models are simple (they constitute either 5 species with 5 interactions or 6 species and 6 interactions), the resulting dynamics are rich. While the effectiveness and toxicity of drug in systems with monotonic input-output relations can typically be predicted intuitively, the behavior of non-monotonic response systems are much more difficult to interpret and in such systems, the response has to be studied over a wide range of conditions (e.g., drug concentration range), and that our intuition can be misleading: For example, and observed decrease in effectiveness and/or increase in toxicity upon increasing drug dose may not imply that a higher drug dose is even worse, as it would be for systems with monotonic input-output relations. In fact, the qualitative behavior of incoherent feed-forward and multiplexing motifs often does not follow from the network architecture alone, but also depends on the quantitative details of the interactions. We find that certain pharmacological manipulations are inherently toxic, and neither single-target nor polypharmacology strategies can be taken to avoid the toxicity. Our study reveals that the optimal drug dose for a certain pharmacological manipulation may be far beyond the onset of toxicity. We have shown that the therapeutic windows for certain therapies are narrow and the preferred concentration range might occur after a broad toxic regime, suggesting that for a newly designed drug, one has to explore administration parameter space carefully to detect the effectiveness of the drug. This is important because apparent dose limitations imposed by toxicity likely discourage clinical trials exploring higher dosage levels and therefore the effectiveness of the drug will remain unnoticed due to inadequate exposure. Clearly, the precise size of a therapeutic window depends on the details of the system of interest, and must be determined by experiment. Our results imply that to determine its size, a much larger parameter scan (e.g., drug concentration range) may be warranted than typically employed hitherto.

## Methods

### Modeling the networks

In Fig. 1 we introduce the 5-node and 6-node networks taken into consideration. We distinguish between two types of interactions, up-regulatory (activation) and down-regulatory (repression). To model these interactions we use the MWC model. Using this statistical physics model we determine the probability of a component to be in the active form. A key concept of the MWC model is that each component can always be either in the active or in the inactive form, independent of the interaction with other components. However, the interaction (e.g. through binding) of another component can shift the balance to either the active or the inactive form.

The probability *p*^*A*^ for a component to be in the active form is computed from the partition functions, which enumerate all possible ways a receptor can be in either the active (*Z*^*A*^) or inactive (*Z*^*I*^) form:


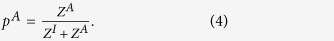


The explicit forms of the partition functions under the MWC model are presented in the Supplementary Material. For intuition, we provide an example here: the partition functions for a component X that is under the regulation of V are


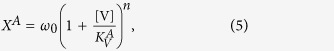



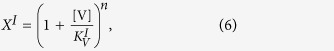


where the parameter 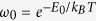 is the Boltzmann factor corresponding to the energy difference *E*_0_ between the active and inactive form, and the parameters 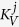 are the dissociation constants of V in form *j* ∈ {*A*,*I*}. In Eq. (5) , the two terms correspond to the component being active when X is unbound and when X is bound. The factor *n* reflects the cooperativity of *n* V components interacting. The same holds for Eq. (6) with the receptor being inactive. Importantly, if 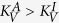, the component V represses X, since V is more likely to bind in the inactive form.

Combining Eq. (4) and Eqs. (5,6), the total concentration of active components X^*A*^ is


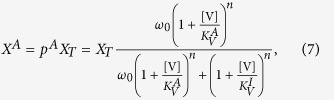


where *X*_*T*_ is the total concentration of X. In the SI we connect the MWC-model to the more common Hill-function formalism.

### Time dependence of drug-administration

The drug concentration, assuming exponential dilution over time, is


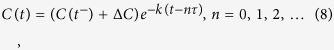


where *C*(*t*^−^) is the concentration at *t*^−^ = *nτ*, just before drug administration, Δ*C* is the administered dose at *t* = *nτ* and *k* is the dilution rate. We assume periodicity on the interval *τ*, meaning that the concentration just before adding Δ*C*, *C*(*t*^−^) equals the concentration after dilution of the dose Δ*C* over a time window *τ*. We thus obtain the concentration just before drug administration (*t*^−^) and directly after drug administration (*t*^+^)


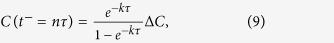



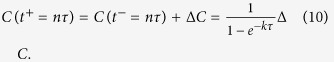


Combining Eqs. (8, 9, 10), we obtain an expression for the dynamics of the drug
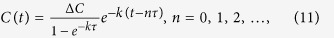


Further we require that the average drug concentration in the time interval [*nτ* : (*n* + 1)*τ*] is *C*_0_ (note that the total administered dose is *C*_*T*_ = *τC*_0_),


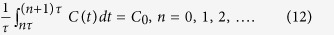


Combining Eq. (11) and Eq. (12) , we write for the drug concentration as a function of time


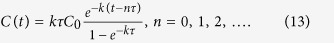


As a result, the maximum and minimum concentration in the time interval [*nτ* : (*n* + 1)*τ*] are


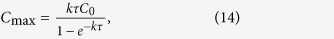



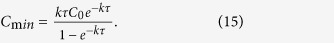


**Figure 7:**
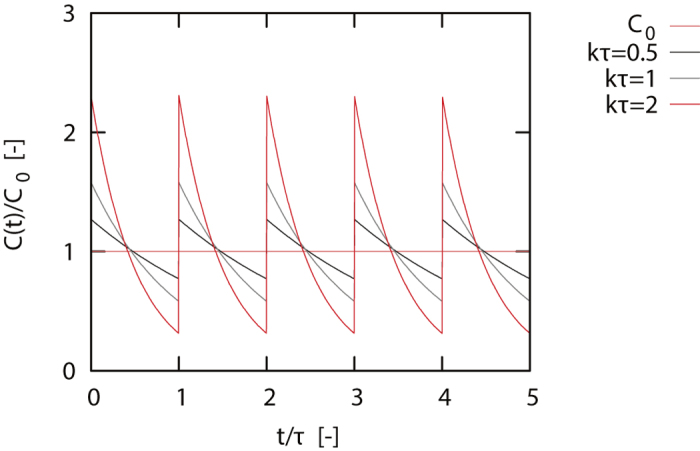
Time dependence of the agonist concentration for different average dose levels *C*_0_. Longer interval times between administration or faster dilution lead to much larger fluctuations in the dose-level.

We define a new parameter *K* = *kτ*, which combines the effect of the dilution rate *k* and dose-interval *τ*, reflecting that fast dilution (large *k*) has a similar effect on the results as a long time-interval (large *τ*). In Fig. 7 we show the agonist concentration as a function of time. The dependence on the time-dilution parameter *K* is clearly visible, where for longer intervals/faster dilution an increase in both the maximum and the minimum drug concentration is observed.

## Additional Information

**How to cite this article**: van Wijk, R. *et al.* Non-monotonic dynamics and crosstalk in signaling pathways and their implications for pharmacology. *Sci. Rep.* **5**, 11376; doi: 10.1038/srep11376 (2015).

## Supplementary Material

Supplementary Information

## Figures and Tables

**Table 1:** Summarized overview of the effects of agonists for the signals S _1_,S_2_, and agonists/antagonist for V.

S_1_	S_2_	Transition	Goal	Result	
OFF	OFF → ON	■ → ▲	X_2_: OFF → ON	Effective, non-toxic	
ON	OFF → ON	▼ → ●	X_2_: OFF → ON	Effective, non-toxic	
OFF → OFF	ON	■ →▼	X_1_: OFF → ON	Effective, **toxic**	
OFF → ON	ON	▲ → ●	X_1_: OFF → ON	Effective, non-toxic	
S_1_	S_2_	**State**	**Goal**	**Transition of V**	**Result**
OFF	OFF	■	X_1_: OFF → ON	■ → ●	Effective, **toxic**
OFF	OFF	■	X_2_: OFF → ON	■ → ▲	Effective, non-toxic
OFF	ON	▲	X_1_: OFF → ON	▲ → ●	Effective, non-toxic
OFF	ON	▲	X_2_: ON → OFF	▲ → ■	Effective, non-toxic
OFF	ON	▲	X_2_: ON → OFF	▲ → ▼	Effective, **toxic**
