# Supplementary material for: Non-monotonic dynamics and crosstalk in signaling pathways and their implications for pharmacology
Source: Sci Rep. 2015 Jun 18;5:11376. doi: 10.1038/srep11376 (PMC5155565; doi:10.1038/srep11376)
Supplement: Supplementary Information [file srep11376-s1.pdf]

SUPPORTING MATERIAL: Non-monotonic dynamics and crosstalk in signaling pathways and their implications for pharmacology

Roeland van Wijk, Sander Tans, Pieter Rein ten Wolde, Alireza Mashaghi

April 29, 2015

[1]Corresponding author: Alireza Mashaghi; Email: alireza_mashaghi-tabari@meei.harvard.edu

SI. 1 Parameter sampling

In this section we describe the parameter sampling which is performed to obtain Fig. 3. First we describe the different partition functions, and second we describe the procedure to sample parameters.

SI. 1.1 Partition functions

In the first section of the methods we describe the general MWC-model. Here we provide the expressions for the partition functions for components that are regulated by 1, 2 or 3 other component(s). For regulation by a single component, the partition functions are equal to the the following expressions:

$$Z^A = \omega_0 \left(1 + \frac{[\mathrm{Z}]}{K_Z^A}\right)^n, \tag{SI-1}$$

$$Z^I = \left(1 + \frac{[\mathrm{Z}]}{K_Z^I}\right)^n, \tag{SI-2}$$

where if $K_Z^A > K_Z^I$ component Z is repressing, otherwise it is activating. For regulation by two components $\mathrm{Z}_1, \mathrm{Z}_2$,

$$Z^A = \omega_0 \left(1 + \frac{[\mathrm{Z}_1]}{K_{Z_1}^A} + \frac{[\mathrm{Z}_2]}{K_{Z_2}^A}\right)^n, \tag{SI-3}$$

$$Z^I = \left(1 + \frac{[\mathrm{Z}_1]}{K_{Z_1}^I} + \frac{[\mathrm{Z}_2]}{K_{Z_2}^I}\right)^n. \tag{SI-4}$$

Constructing the partition functions in this way, implies that both Z_1 and Z_2 have independent binding locations and do not (structurally) influence the other. Generalizing the above partition functions for k components Z_i

$$Z^A = \omega_0 \left(1 + \sum_{i=1}^{k} \frac{[\mathrm{Z_i}]}{K_{Z_i}^A}\right)^n, \tag{SI-5}$$

$$Z^I = \left(1 + \sum_{i=1}^{k} \frac{[\mathrm{Z_i}]}{K_{Z_i}^I}\right)^n. \tag{SI-6}$$

SI. 1.2 Parameter space

For the signal range we use $S = [\mathrm{s_{min}} = 1, 2, 5, 10, 20, 50, 100, 200, 500, \mathrm{s_{max}} = 1000]$ $[au]$. We then generate a random initial condition, using the following parameter constraints for the dissociation constants K_D

- $K_D^{S_i}$: uniformly sampled between $\left[10^{-1} : \times 10^4\right]$
- $K_D^{\{V,W,X_1\}}$: uniformly sampled between $\left[10^{-1} : 10^4\right]$
- $\omega^{\{V,W,X_1,X_2\}}$: uniformly sampled between $\left[5 \times 10^{-2} : 2 \times 10^1\right]$

- Only for 5 node topology: $\omega^{\{V\}}$: uniformly sampled between $\left[5 \times 10^{-3} : 10^{3}\right]$

In the second step the network is optimized using a simulated annealing algorithm to suffice the following conditions

1. For $S_2 = 0.1$, an increase in S_1 from $\mathrm{s}_{\mathrm{min}} \rightarrow \mathrm{s}_{\mathrm{max}}$ results in a change in the form of X_1 from ON/OFF to OFF/ON

2. For $S_1 = 0.1$, an increase in S_2 from $\mathrm{s}_{\mathrm{min}} \rightarrow \mathrm{s}_{\mathrm{max}}$ results in a change in the form of X_2 from ON/OFF to OFF/ON

In other words, only networks for which each response changes state for an increase in its respective signal are taken into consideration. For this reason, not every topology has the same number of parameter sets, since for some topologies the parameter space that encompasses these constraints is much smaller.

In Fig. SI-1 we show the influence of the parameters α and n on the results. As expected, an increase in α reduces the number of topologies that show non-monotonic behavior, and has a more pronounced effect on topologies with only coherent feed-forward interactions. A decrease in the Hill-coefficient n has a similar effect, since this reduces the steepness of the dose-response curve and therefore flattens the effect of each component.

SI. 1.3 Implications of the MWC model

Interestingly, the widely used MWC model has some interesting consequences for our model. One of the consequences, which already is indicated in the main text is that coherent feed-forward loops can show a non-monotonic response. The origin of this behavior lies in potential saturation effects. For clarity, the example from the main text is further illustrated in Fig. SI-2. Indeed, in our model, the concentration of $\mathrm{V}, \mathrm{X}_1, \mathrm{X}_2$ and W are constant on the timescale of our interest.

Looking closely at Eq. SI-3 it is clear that if $K^A_{Z_1} > K^A_{Z_2}$ the partition function is dominated by $Z_2/K^A_{Z_2}$ at equal concentration $Z_1 = Z_2$. However, for many biological processes the total amount of a specific component is bounded completely, for example in a simple process where a protein is phosphorylated and the total concentration of protein stays constant; or the concentration of protein remains constant for some time, since the production of new protein is a time consuming process. However, if saturation effects are important, the behavior of the partition function can for be dominated by both proteins, even if the dissociation constants are very different.

If $Z_1(Y)$ and $Z_2(Y)$ are both function of Y, but Z_2 saturates at much smaller values of Y, then for small Y the increase in the partition function is dominated by Z_2, while for large Y the increase in the partition function is dominated by Z_1.

There is another consequence; which is especially important for the multiplexing motif. Assume that an incoherent feed-forward loop exists between V, X_1 and X_2, where V regulates X_1 and X_2 positively, while X_1 represses X_2. As a result the dose-response curve of X_1 as function of V is monotonically increasing. What can we say about the dose-response curve for X_2 as function of V?

For increasing V, X_2 increases. This increase is repressed by the increase in X_1. The dose response curve then will be a concave function, increasing for small V and decreasing at larger V. But, at some concentration of V, X_1 saturates, leading to a saturated repression strength. Even before saturation, the increase in X_1 as function of increasing V is marginal, as is the repression strength. However, an increase in V leads to an increase in X_2; if the increase in V is large enough,

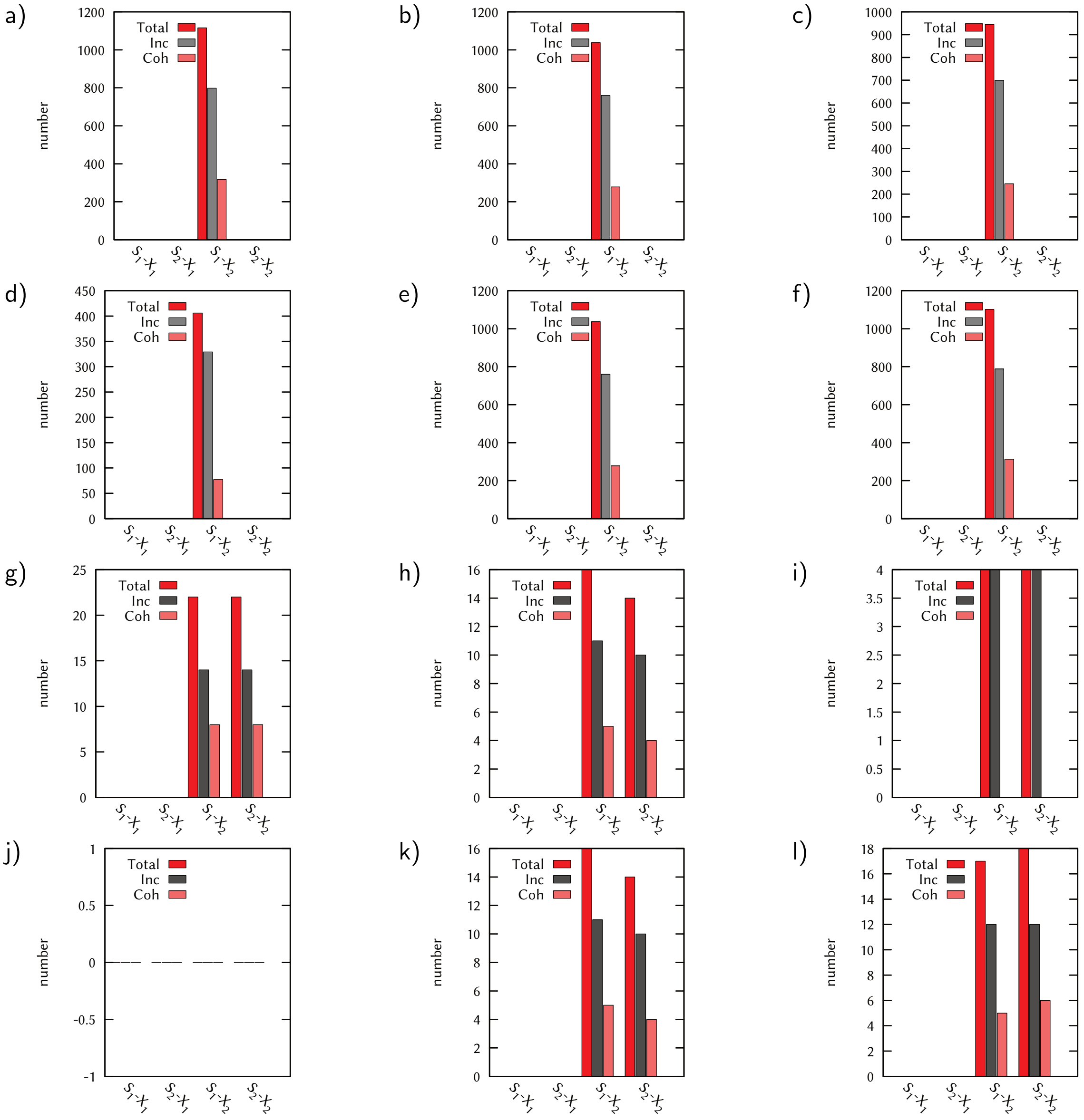

Figure SI-1: a,b,c: 6-node network with $n = 2$; respectively $\alpha = 0.55, 0.65, 0.75$. d,e,f: 6-node network with $\alpha = 0.65$; respectively $n = 1, 2, 3$. g,h,i: 5-node network with $n = 2$; respectively $\alpha = 0.55, 0.65, 0.75$. j,k,l: 5-node network with $\alpha = 0.65$; respectively $n = 1, 2, 3$.

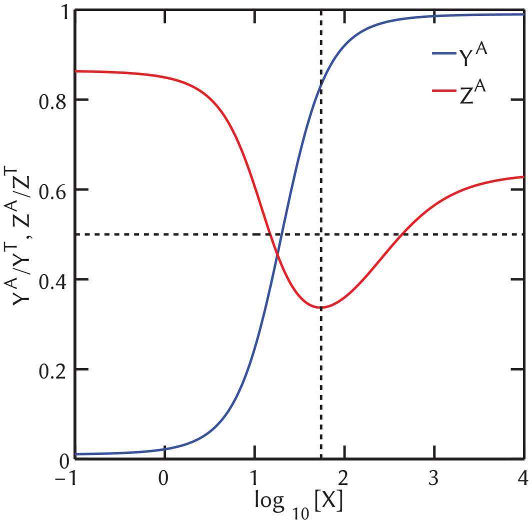

Figure SI-2: Figure illustrating non-monotonic behavior in a coherent feed-forward loop as explained in the main text on page 4. Plotted are the the active fraction of component Y and Z as a function of the concentration of component X. $\alpha = 0.5$ is indicated by the horizontal dotted line. The vertical dotted line indicates from which point Y starts to saturate, while X is still increasing. Parameters used are: $n = 2$, $\omega_Y = 0.01[k_BT]$, $\omega_Z = 7[k_BT]$, Ytotal = Ztotal = 2000 [au], $K^A_{XY} = 2[\mu\text{M}]$, $K^I_{XY} = 200[\mu\text{M}]$, $K^A_{XZ} = 100[\mu\text{M}]$, $K^I_{XZ} = 50[\mu\text{M}]$, $K^A_{YZ} = 12000[\mu\text{M}]$ and $K^I_{YZ} = 400[\mu\text{M}]$.

this overcomes the repression and X_2 rises again. We thus obtain a complicated dose-response relation for X_2 as function of V. For small and large V, X_2 rises, while for intermediate values, X_2 decreases. This behavior is a direct consequence of the MWC-model, combined with the saturation of the components that are the constituents of the model.

SI. 2 Analytical proof

In this short section we show that non-linear dependencies on the regulating components is required to obtain non-monotonic behavior. For a non-monotonic response of e.g. Y a necessary requirement is that the derivative of $p_Y^A = Z^A/(Z^I + Z^A)$ with respect to one of the regulatory components changes sign, which means that

$$\frac{dp_Y^A}{dZ_i} = \frac{Z^I \frac{dZ^A}{dZ_i} - Z^A \frac{dZ^I}{dZ_i}}{\left(Z^I + Z^A\right)^2} \tag{SI-7}$$

and since the denominator is always larger than zero, we only have to focus on the numerator. As an example, we take a model for component Y with regulatory components Z_1 and Z_2, and arbitrary n, and look for non-monotonic behavior as function of Z_1. Thus we obtain

$$Z^I \frac{dZ^A}{dZ_i} - Z^A \frac{dZ^I}{dZ_i} = \omega_0 \frac{Z^I}{K_{Z_1}^A} - \frac{Z^A}{K_{Z_1}^I} \tag{SI-8}$$

$$= g\left(Z_1, Z_2\right) \left[\frac{1 + \frac{Z_1}{K_{Z_1}^I} + \frac{Z_2}{K_{Z_2}^I}}{K_{Z_1}^A} - \frac{1 + \frac{Z_1}{K_{Z_1}^I} + \frac{Z_2}{K_{Z_2}^I}}{K_{Z_1}^I}\right] \tag{SI-9}$$

$$= g\left(Z_1, Z_2\right) \left(\frac{1 + \frac{Z_2}{K_{Z_2}^I}}{K_{Z_1}^A} - \frac{\omega_0 \left(1 + \frac{Z_2}{K_{Z_2}^I}\right)}{K_{Z_1}^I}\right), \tag{SI-10}$$

where $g(Z_1, Z_2) = n\omega_0 \left(\left(1 + \frac{Z_1}{K_{Z_1}^I} + \frac{Z_2}{K_{Z_2}^I}\right)\left(1 + \frac{Z_1}{K_{Z_1}^A} + \frac{Z_2}{K_{Z_2}^A}\right)\right)^{n-1}$. Since $g(Z_1, Z_2)$ is either positive or negative as function of Z_1, the sign of the derivative is not dependent on Z_1. This result is different if Z_2 is a non-linear function of Z_1, as is the case in feed-forward loops. In the same framework of the MWC-model, Z_2 is

$$Z_2^A\left(Z_1\right) = Z_2^T \frac{\omega_0 \left(1 + \frac{Z_1}{R^A}\right)}{\omega_0 \left(1 + \frac{Z_1}{R^A}\right) + 1 + \frac{Z_1}{R^I}}, \tag{SI-11}$$

with R^j the dissociation constants for the regulatory component Z_1 with respect to Z_2. A straightforward derivation now shows that the sign of the derivative of p_Y^A does depend on Z_1 and as a result non-monotonic behavior could be observed.

SI. 3 Pharmacology of multiplexing model

SI. 3.1 A more in depth discussion of the multiplexing motif

The multiplexing motif (Fig. SI-3a) is a signaling motif that is capable of multiplexing two input signals — S_1 and S_2 — into two signal dependent responses — X_1 and X_2 — while sharing intermediate component(s) (1). For biologically relevant parameters, this topology can lead to a monotonic relation between S_1 and X_1 exists, and similarly also a monotonic relation between S_2

and X_2 exists (Figs. 5b,c). The state of X_1 is only dependent on the state of S_1, but not on the state of S_2.

This motif is capable of multiplexing two signals with two input states; S_i is either `ON` or `OFF`. Since each signal has two states, in total four input states (■, ▲, ▼, •) exist (see Table. SI-1), which map to four shared concentration V^i, and four output concentrations X_i^j. Importantly, for the output concentrations X_i, we define X_i^0, X_i^1, the two lowest concentrations to be the `OFF`-state, and X_i^2, X_i^3 as the `ON`-state, such that each response is characterized by being in the `ON` or `OFF`-state (Fig 5a).

In the multiplexing motif both S_1 and S_2 activate V via competitive binding — like two ligands activating a receptor with different dissociation constant, leading to a non-trivial relation for V on its input signals (Fig 5a). Indeed, as function of S_1, V is a monotonically decreasing function (Fig. SI-3b), independent of the state of S_2. However, as function of S_2, V is either monotonic increasing or decreasing, depending on the actual state of S_1 (Fig. SI-3b).

V activates X_1 and represses X_2, while X_1 activates X_2, creating an incoherent feed-forward loop between V, X_1 and X_2 (2; 3). Due to the incoherent feed-forward loop, X_2 is a non-monotonic function of V (Fig 5a). Indeed, X_1 activates X_2, but due to the repression from V, the activation by X_1 is canceled, leading to an effective decrease. Importantly, the combined effect of the non-monotonic relations between both V and S_2 and between X_2 and V results in a non-intuitive monotonic relation between X_2 and S_2.

We define three critical concentrations for V that determine the change of the state of X_1 or X_2. For $V > V^*_{X_1}$, X_1 is in the `ON`-state, while for $V^*_{X_2} < V < V^{*,\gamma}_{X_2}$, X_2 is in the `ON`-state. These critical concentrations are indicated in (Fig 5a). To allow for multiplexing, the following relation between the critical concentration should hold $V^*_{X_2} < V^*_{X_1} < V^{*,\gamma}_{X_2}$ (1).

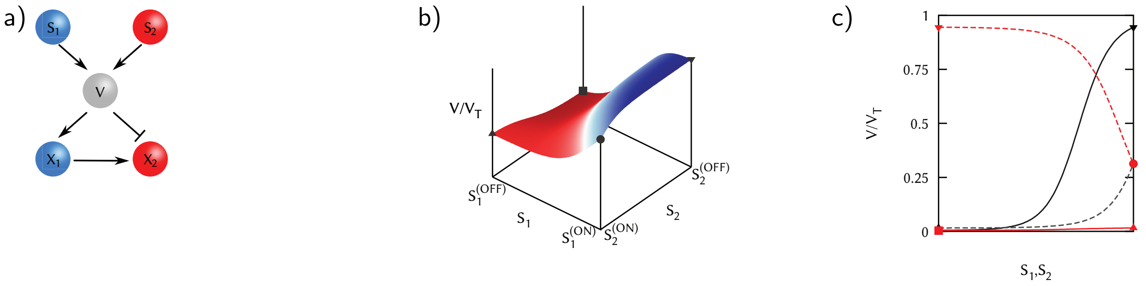

Figure SI-3: a) Multiplexing motif b) Dose-response relation between V and S_1 and S_2. c) Dose-response curves between V and S_1 (black) for S_2 `OFF` (black solid) and `ON` (black dashed), and V and S_2 (red) for S_1 `OFF` (red solid) and `ON` (red dashed). Symbols correspond to specific states of the input signals (see Table. SI-1).

SI. 3.2 Parameters used in the main text

In this section we provide the parameters of the 5 and 6 node model used in the main text.

Table SI-1: Overview of the different initial states with corresponding concentration levels of V, X_1 and X_2.

State	Symbol	S_1	S_2	V	X_1	X_2
A	■	OFF	OFF	V^1	X_1^0	X_2^3
B	▲	OFF	ON	V^2	X_1^1	X_2^0
C	▼	ON	OFF	V^3	X_1^3	X_2^1
D	●	ON	ON	V^2	X_1^2	X_2^2

Table SI-2: Parameter values 6-node model

**Parameter**	**Value**	**Parameter**	**Value**
$\omega_{0,V}$	5.0000×10^{-2} [k_BT]	V_T	2000
$K_{V,A}^{S_1}$	3.70165×10^{1} [μM]	$K_{V,I}^{S_1}$	1.28135×10^{3} [μM]
$\omega_{0,W}$	1.75584×10^{-1} [k_BT]	W_T	2000
$K_{W,A}^{S_2}$	6.68111×10^{1} [μM]	$K_{W,I}^{S_2}$	1.30206×10^{3} [μM]
ω_{0,X_1}	5.52685×10^{-2} [k_BT]	$X_{1,T}$	2000
$K_{X_1,A}^{V}$	4.28908×10^{1} [μM]	$K_{X_1,I}^{V}$	1.01301×10^{3} [μM]
ω_{0,X_2}	4.74499×10^{-1} [k_BT]	$X_{2,T}$	2000
$K_{X_2,A}^{V}$	5.50058×10^{2} [μM]	$K_{X_2,I}^{V}$	1.2969×10^{2} [μM]
$K_{X_2,A}^{W}$	2.15566×10^{3} [μM]	$K_{X_2,I}^{W}$	4.88937×10^{2} [μM]
$K_{X_2,A}^{X_1}$	1.48882×10^{2} [μM]	$K_{X_2,I}^{X_1}$	1.71212×10^{3} [μM]

Table SI-3: Parameter values 5-node model

**Parameter**	**Value**	**Parameter**	**Value**
$\omega_{0,V}$	3.4800×10^{-3} [k_BT]	V_T	2000
$K_{V,A}^{S_1}$	8.2608×10^{-1} [μM]	$K_{V,I}^{S_1}$	1.3820×10^{2} [μM]
$K_{V,A}^{S_2}$	4.0298×10^{0} [μM]	$K_{V,I}^{S_2}$	9.0000×10^{0} [μM]
ω_{0,X_1}	1.0000×10^{-2} [k_BT]	$X_{1,T}$	2000
$K_{X_1,A}^{V}$	3.8156×10^{0} [μM]	$K_{X_1,I}^{V}$	3.8199×10^{2} [μM]
ω_{0,X_2}	2.0075×10^{-3} [k_BT]	$X_{2,T}$	2000
$K_{X_2,A}^{V}$	1.8340×10^{1} [μM]	$K_{X_2,I}^{V}$	1.5569×10^{1} [μM]
$K_{X_2,A}^{X_1}$	8.3599×10^{-1} [μM]	$K_{X_2,I}^{X_1}$	5.8491×10^{2} [μM]

SI. 3.3 Extension of Table 1 from the main text

In this section we provide the complete Table with all possible initial states and the possible agonist/antagonist for S_1, S_2, and V. Table. SI-4 provides all the required information on the effectiveness and toxicity of drugs.

S_1	S_2	**Transition**	**Goal**	**Result**	
OFF	OFF→ON	■ → ▲	X_2: ON→OFF	Effective, non-toxic	
ON	OFF→ON	▼ → ●	X_2: ON→OFF	Effective, non-toxic	
OFF	ON→OFF	▲ → ■	X_2: OFF→ON	Effective, non-toxic	
ON	ON→OFF	● → ▼	X_2: OFF→ON	Effective, non-toxic	
OFF→ON	OFF	■ → ▼	X_1: OFF→ON	Effective, **toxic**	
OFF→ON	ON	▲ → ●	X_1: OFF→ON	Effective, non-toxic	
ON→OFF	OFF	▼ → ■	X_1: ON→OFF	Effective, **toxic**	
ON→OFF	ON	● → ▲	X_1: ON→OFF	Effective, non-toxic	
S_1	S_2	**State**	**Goal**	**Transition of V**	**Result**
OFF	OFF	■	X_1: OFF→ON	■ → ●	Effective, **toxic**
OFF	OFF	■	X_2: ON→OFF	■ → ▲	Effective, non-toxic
OFF	ON	▲	X_1: OFF→ON	▲ → ●	Effective, non-toxic
OFF	ON	▲	X_2: OFF→ON	▲ → ■	Effective, non-toxic
OFF	ON	▲	X_2: OFF→ON	▲ → ▼	Effective, **toxic**
ON	OFF	▼	X_1: ON→OFF	▼ → ▲	Effective, **toxic**
ON	OFF	▼	X_2: ON→OFF	▼ → ●	Effective, non-toxic
ON	ON	●	X_1: ON→OFF	● → ▲	Effective, non-toxic
ON	ON	●	X_2: OFF→ON	● → ▼	Effective, non-toxic
ON	ON	●	X_2: OFF→ON	● → ■	Effective, **toxic**

Table SI-4: Total overview of the effects of agonists for the signals S_1, S_2, and agonists/antagonist for V (see also Fig. 6 and Table. SI-1).

SI. 3.3.1 Influence of differences between individuals for the 5 node model

Here we show similar results for the 5 node model as in the paragraph in the main text for the 6 node model for differences in the kinetic parameters between individuals. Since our analysis depends on specific dissociation constants, both for the signals S_1 and S_2 to V and for V to X_1 and X_2, we study the influence of these variations on the effectiveness and toxicity of an antagonist.

Assuming we start in state ▼ or ■, we study the effect of the application of an agonist for respectively S_2 (intending to move the system to state ●) or for V (intending to move the system to state ▲). We take for the administration parameter $K = 0.1$, and use the average dose C_0 as the variable of interest (we thus take a horizontal cut of the contour plot at $\log_{10}[K] = -1$ in Figs. 8a,b. We mimic the intrinsic variations following Eq. 6 in the main text.

An agonist for S_2 (Fig. SI-4a) has for increasing λ (increasing the variability) a decreasing dose-range of 100% effective and non-toxic dose. In other words, a higher variability leads to a more narrow window that is effective for all individuals. However, the dose-range for which only a small fraction of individuals has an effective treatment increases. This is due to the fact that larger differences between individuals exist, and the probability that a relative high/low concentration works for a single individual increases. Remarkably, the 50% crossing is almost constant for all λ. In Fig. SI-4b a similar dependence on the variability for an agonist for V is shown.

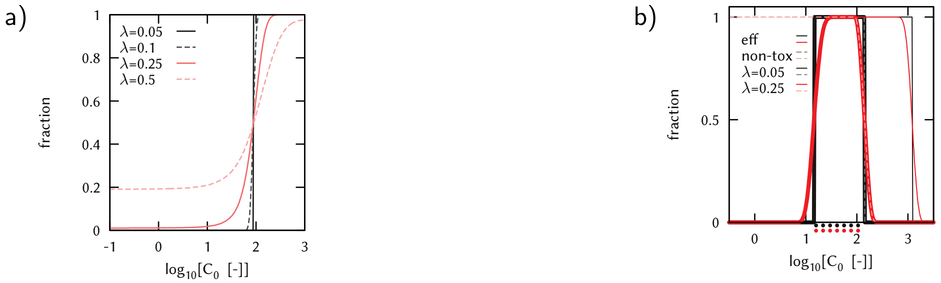

Figure SI-4: The effect of intrinsic variability. A relative change λ in all the intrinsic parameters (see Eq. 6) leads to a reduction of the effectiveness and/or an increase in the toxicity. Shown is the fraction of parameter combinations for which a drug, with administration dynamics $K = 0.1$, as a function of C_0 is effective and non-toxic. a) Lines show for an agonist for S_2 (with S_1 in state `ON`) the fraction of individuals for which — at the given dose C_0, and $K = 0.1$ — an effective and non-toxic treatment is obtained. b) Similar results for an agonist for V, however the results for effectiveness (thin lines) and non-toxicity (thick lines) are shown separately. The thick dotted lines denotes the therapeutic window for $\log_{10}[C_0] \approx [1.2 : 2.15]$. $n = 2, \alpha = 0.55$.

SI. 3.3.2 Dependence of the therapeutic window on parameter settings in the 6 node network.

In this section we show the effect of intrinsic variability in the 6 node network from Fig. 1b.3 in the main text for a different parameter setting, namely $\alpha = 0.55$. This change in α shows a larger therapeutic window due to a large change in the fraction of non-toxic and effective cells as shown in Fig. SI-5b.

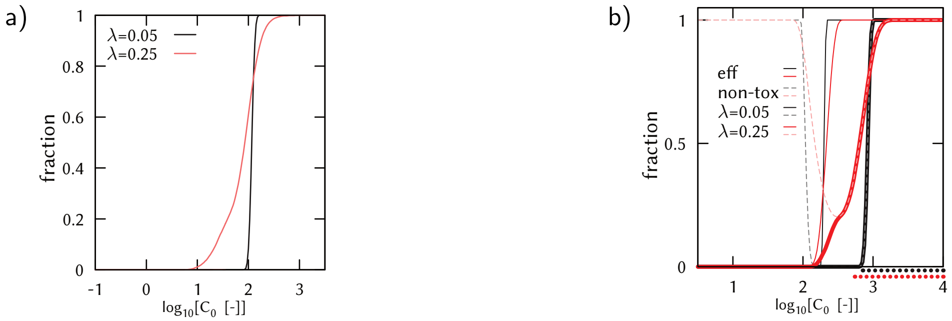

Figure SI-5: The effect of intrinsic variability in the 6 node network from Fig. 1b.3 in the main text. $\alpha = 0.55$, $n = 2$. A relative change λ in all the intrinsic parameters (see Eq. 2 in the main text) leads to a reduction of the effectiveness and/or an increase in the toxicity. Shown is the fraction of parameter combinations for which a drug with concentration C_0 is effective and non-toxic. a) Lines show for an agonist for S_1 (with $S_2 = 200$) the fraction of individuals for which — at the given dose C_0 — an effective and non-toxic treatment is obtained. b) Similar results for an agonist for V, however the results for effectiveness (thin lines) and non-toxicity (thick lines) are shown separately. The solid thick lines indicates the dose for which the drug is non-toxic and effective . The dotted thick lines denotes the therapeutic window $(S_1 = 0.1, S_2 = 200)$.

Supporting References

[1] de Ronde, W., Tostevin, F. & ten Wolde, P. R. Multiplexing biochemical signals. *Phys. Rev. Lett.*, **107,** 1–4, (2011).

[2] Shen-Orr, S. S., Milo, R., Mangan, S. & Alon, U. Network motifs in the transcriptional regulation network of Escherichia coli *Nat. Gen.*, **31,** 64–68, (2002).

[3] Yeger-Lotem, E., Sattath, S., Kashtan, N., Itzkovitz, S., Milo, R., Pinter, R. Y. Alon, U. & Margalit, H. Network motifs in integrated cellular networks of transcription-regulation and protein-protein interaction. *P. Natl. Acad. Sci. USA*, **101,** 5934–5939, (2004).
